# Current practices in cancer spatial data analysis: a call for guidance

**DOI:** 10.1186/1476-072X-4-3

**Published:** 2005-01-13

**Authors:** Linda Williams Pickle, Lance A Waller, Andrew B Lawson

**Affiliations:** 1 Division of Cancer Control and Population Sciences, National Cancer Institute, 6116 Executive Blvd., Suite 504, Bethesda, MD 20892, USA; 2 Department of Biostatistics, Rollins School of Public Health, 1518 Clifton Road NE Atlanta, GA 30322, USA; 3 Department of Biostatistics and Epidemiology, Arnold School of Public Health, University of South Carolina, Columbia, SC, USA

## Abstract

There has long been a recognition that place matters in health, from recognition of clusters of yellow fever and cholera in the 1800s to modern day analyses of regional and neighborhood effects on cancer patterns. Here we provide a summary of discussions about current practices in the spatial analysis of georeferenced cancer data by a panel of experts recently convened at the National Cancer Institute.

## Review

### Background

Recently, the North American Association of Central Cancer Registries (NAACCR) formed a Geographic Information Systems (GIS) Task Force that prepared a handbook to aid cancer registry staff in using GIS for the collection, analysis, and presentation of cancer registry data [1]. The first chapters of the NAACCR handbook provide extensive information on registry data issues, particularly address geocoding and confidentiality. In June, 2002, the National Cancer Institute sponsored a meeting of selected subject matter experts in Bethesda, MD, to expand the analytic overview in the NAACCR effort to focus specifically on spatial data analysis. Invitees (listed in Table 1) include individuals with backgrounds in statistics, epidemiology, and geography so as to balance the points of view expressed.

**Table 1:** Panel members, home institutions, and self-selected focus areas for break-out discussions. The following lists all panel members, their home institutions, and each member's top choices of topics for break-out discussions. All panel members contributed significantly to the general discussion and to initial break-out discussions. A subset of panel members expanded on initial discussions to create the reports listed in Table 2.

**Name**	**Institution**	**Primary topics of collaboration**
Luc Anselin	University of Illinois, Urbana-Champaign	Spatial computing, spatial analysis, and exploratory spatial data analysis
B. Sue Bell	National Cancer Institute, (currently, Food and Drug Agency)	Communicating the results of spatial health analyses, features of spatial data, and disease surveillance
Francis Boscoe	New York State Department of Health	Features of spatial data, exploratory data analysis, and limitations of spatial analysis
Barnali Das	National Cancer Institute	Spatial modeling, exploratory spatial data analysis, and spatial cluster detection.
Carol Gotway	Centers for Disease Control and Prevention	Exploratory spatial data analysis, spatial modeling, and features of spatial data
William Henriques	Agency for Toxic Substances and Disease Registry	Features of spatial data, overview of spatial analysis, and communicating results of spatial health analyses
Theodore Holford	Yale University	Disease surveillance, spatial modeling, and exploratory spatial data analysis
Richard Hoskins	Washington State Department of Health	Communicating the results of spatial health analyses, overview, and spatial computing
Geoffrey Jacquez	Biomedware	Limitation of spatial analyses, spatial cluster detection, and overview of spatial analysis.
Martin Kulldorff	Harvard	Exploratory spatial data analysis, spatial cluster detection, and disease surveillance
Andrew Lawson	University of South Carolina	Overview of spatial analysis, and spatial cluster detection.
Linda W. Pickle	National Cancer Institute	Project coordinator, overview of spatial analysis, communication of spatial health analyses, spatial modeling, and exploratory spatial data analysis
Peggy Reynolds	Environmental Health Investigations Branch, California State Department of Health	Spatial modeling, features of spatial data, and disease surveillance
Gerard Rushton	University of Iowa	Exploratory spatial data analysis, features of spatial data, and spatial modeling
Lance Waller	Emory University	Chair of panel, spatial modeling, spatial cluster detection, and overview of panel discussion
Mary Ward	Division of Cancer Epidemiology and Genetics, National Cancer Institute	Features of spatial data, disease surveillance, and overview of spatial analysis
Dan Wartenberg	University of Medicine and Dentistry of New Jersey	Spatial cluster detection, exploratory spatial data analysis, and communicating the results of spatial health analyses
Dale Zimmerman	University of Iowa	Spatial modeling, spatial cluster detection, and exploratory spatial data analysis

The purpose of the meeting was to provide guidance from experts in this field who have experience applying these methods to health data, acknowledging that opinion will change as the field continues to evolve. Consensus of recommendations for any technical field is difficult to achieve, but we have attempted to include contributors with a wide-ranging set of backgrounds and experiences in the hope that what is presented represents, if not clear "best practices", at least sound principles for the analysis of spatial health data. This paper introduces motivating ideas and provides a broad overview of an upcoming series of reports by subgroups of the attendees. A listing of initial reports appears in Table 2, and additional topic-specific reports are in preparation.

**Table 2:** Titles and authors of initial reports by panel members (drafts available upon request). These reports represent summaries and expansions of initial discussions by the panels. The author team took topics and ideas generated by the panel discussions, conducted literature searches, formalized the presentation structure and composed the report. The final reports represent the collective efforts of each author team, building on selected contributions of panel members.

Title	Author Team	Topics
Current practices in cancer spatial data analysis: a call for guidance	Linda W. Pickle, Lance Waller, Andrew Lawson	Introduction to panel discussion and background issues.
Communication: reporting spatial health statistics to policy makers and the general public	B. Sue Bell, Richard E. Hoskins, Daniel Wartenberg	Review of issues involved in communicating results of spatial analyses of cancer data.
Current practices in spatial analysis of cancer data: data characteristics and data sources for geographic studies of cancer	Francis P. Boscoe, Mary H. Ward, Peggy Reynolds	Review of characteristics and sources of spatially-referenced health data.
Current practices in the spatial analysis of cancer: flies in the ointment	Geoffrey M. Jacquez	Unresolved issues lurking behind most spatial analyses of health data.

### Motivation

Interest in and use of GIS for health data has grown tremendously during the past decade. The recognition of local geographic influences on health date back at least to the development of spot maps of yellow fever and cholera in the earlier-to-mid 1800's [2]. While what is known today as GIS grew out of developments associated with the Canadian Land Inventory in 1963 [3], there were no articles on GIS and human health included in the National Institutes of Heath's (NIH) MEDLINE bibliographic database as recently as 1993; between 1994 and 2002 the number of GIS articles grew 26% per year, four times the rate of increase for human health articles in general. Consequently, the NIH library first added "Geographic Information Systems" as a MEDLINE indexing term in 2003. What has fueled this increased attention? Most attribute it to the increasing computing power and availability of appropriate software on everyone's desktop, thus moving GIS and other analytic tools from the hands of the geographers and computer specialists to those of the health researcher. For example, when the National Cancer Institute prepared its first cancer mortality atlas in the early 1970s [4], the maps had to be prepared on National Oceanographic and Atmospheric Administration computer systems, since they were one of the few government agencies capable of preparing high quality maps. Now anyone with a standard personal computer can prepare such maps on their desktop in just a few minutes. Similarly, complex statistical analyses of georeferenced health data also can run on the desktop. While anyone with access to desktop computing and georeferenced health data can make maps, there is no guarantee that such maps provide meaningful insight to the underlying disease and social processes due to potential epidemiologic, cartographic, and/or statistical issues (e.g., confounding variables, poor choice of visual variables, and/or very small local sample sizes). As a result, the need remains for thoughtful application and appreciation of data, analytic, and interpretive assumptions commonly encountered in the analysis of spatially-referenced health data.

In addition to the impact of the computer revolution is the increasing recognition that all health data are spatial, i.e., referenced to place. A recent call for more widespread use of GIS in the U.K. National Health Service points out that GIS could "act as powerful evidence-based practice tools for early problem detection and solving" [5]. Many health outcomes are related to an individual's "environment" at both the personal and community levels. Personal environmental factors include not only the obvious water, soil, and air content and exposure to hazardous materials, but also lifestyle factors, such as exposure to tobacco smoke (personal and environmental), occupation, transportation choices, hobbies, and characteristics of the home. Community effects, referred to as "neighborhood social context" in the social sciences literature, have been shown to impact health care policy, delivery, utilization and outcomes [6-10]. Even within a specific geographic area, health care often varies among subgroups of residents, leading to the important study of health disparities. As another example, we are just beginning to realize how characteristics of our built environment, such as sidewalks or green space, impact health through relationships to individual's physical activity level [11].

With the increasing interest in and availability of georeferenced health data comes the need for methods to properly analyze them, taking into account the spatial correlation of outcomes in nearby places. Recognition of spatial influences in statistical inference date back to some of the earliest developments of modern statistical methods leading, for example, to notions of randomized plot designs for agricultural field trials [12].

Development of theoretical methods for spatially referenced data includes point process models [13,14], spatial prediction [15,16], and spatial lattice models [17-19] in fields such as agriculture, entomology, bacteriology, cosmology, mining, and meteorology. Methods for the analysis of measurements taken at fixed point locations as random processes grew from independent developments by Matheron [15] and Gandin [16] for analyzing geologic data. While the areas of spatial statistics, statistical computing, and GIS all developed substantially from the 1960's through today, these developments have been and continue to be largely separate and independent of one another.

Application of spatial statistical methods is more common now that both GIS and spatial statistical software packages are widely available. While there are several texts focused on statistical methods for spatial health data [20-25], and health applications of GIS [26-28], there is a growing need for guidance in the combination of the two areas, in particular the selection and proper use of the appropriate statistical techniques for different types of georeferenced health data.

### Complicating factors

We specifically focus on spatial and spatio-temporal statistical methods appropriate for observational human health data, not clinical trials or data from other types of designed experiments. A challenging but common problem with this type of data is the difficulty in obtaining accurate exposure and disease outcome data for the time and place most relevant to that disease. Health consequences are the result of a continuum of multiple and varied exposures which often occur over a long period of time and in various places. How can we capture this complex pattern of exposure over decades and, most important for the topic at hand, to what geographic location do we assign the exposure? A subgroup of meeting attendees discussed the problems of defining "place" and locating appropriate data in detail (see Table 2). A forthcoming article in the International Journal of Health Geographics by Boscoe, Ward, and Reynolds will address these issues.

Another problem with data on human health is that the data required for analysis are typically scattered across many sources and often collected by different groups and agencies. For example, unless we belong to a closed medical system such as a health maintenance organization or military services, each person's medical records are housed in different medical offices. Such records collected for clinical purposes also rarely include demographic information desirable for the data analysis and only include the patient's home address (primarily for billing or other contact purposes), offering no information on previous residences or workplace location(s). Accumulating and validating data required for analysis from these multiple sources usually takes longer than the analysis itself, where data validation plays an essential but time-consuming and often overlooked role.

Of increasing concern in this field is protecting the privacy and confidentiality of the study subjects. While all researchers agree that this is important, it is often difficult to reconcile these needs with data needs for a proper analysis. In particular, spatial data analysis and mapping of results are often hampered by the lack of specific addresses. Data collection agencies and medical facilities are imposing increasingly strict requirements for data release and often only identify a place (usually patient's address) to a broad administrative unit. For example, the recently enacted Health Insurance Portability and Accountability Act (HIPAA) often requires removal of geographic subdivisions smaller than the state, and the National Center for Health Statistics only releases death certificate data aggregated to the county level or for places with large populations. The reportable specificity of location is often not good enough to allow the analysis to answer research questions about the spatial patterns of the disease. Methods are currently being explored that would allow use of specific individual information in the analysis but would mask identifying characteristics in the results reported only at an aggregated level. In addition to such federal reporting restrictions, state and local governments may add additional regulatory requirements.

This said, such regulations need not prohibit spatial analysis of health data completely, rather they change the context within which such analyses may occur. For instance, mutually agreeable memoranda of understanding between analysts and agencies holding data often allow analysis of data with individual-level identifiers provided all reports include only aggregate results. Such memoranda also specify when, if ever, detailed maps of locations may be reported. As an example, the National Cancer Institute's Long Island Breast Cancer Study Project provides a protected environment (on site or remotely accessible) within which registered users may analyze (but not remove) sensitive georeferenced health data (for more information, see ).

In addition to general concerns regarding the analysis of health data, the spatial data analysis of cancer data poses unique challenges. Most cancers develop over a period of 20 to 30 years and are a result of multiple exposures interacting with the individual's genetic susceptibility. Few Americans live in a single place for decades – migration presents the problem of which residential address to use for a case's location. Because latencies differ by cancer type and most likely by an individual's susceptibility, little guidance is available for this question. The rarity of cancer also causes a sparse data problem for analysis, both for detecting clusters in data with high spatial variability and for communication of results without violating confidentiality. John Snow's illustration of his theorized cause of cholera in London via a map of case residences was possible because of the large number of cases in a small geographic area with a single, precisely located exposure [29]. The detection of clusters of a rare disease such as cancer requires sophisticated statistical tools that filter out potentially confounding effects of age, spatially-varying population density, and mobility. As pointed out by Waller and Gotway [25], different statistical methods answer different questions and require care in appropriate application and interpretation. Further discussion of these and other limitations of spatial data analysis are addressed in the accompanying article by Jacquez [30].

Despite such concerns, important discoveries in cancer research do result from spatial data analysis. Although U.S. mortality data had been published in tabular form for many years, it wasn't until mortality rates were mapped in 1975 that spatial patterns emerged, such as the cluster of high oral cancer rates in southeastern states, later found to be due to smokeless tobacco use [4,31]. Later, a number of clusters of childhood leukemia were identified, for example in Seascale, UK, and Toms River, NJ [32,33]. Although environmental, genetic and viral hypotheses have been proposed, the cause of most of these clusters remains unclear [34]. These studies illustrate the potential impact of spatial data analysis on medical research.

Finally, in order to ultimately improve public health, the results of the complex analyses of georeferenced cancer data must be disseminated to those in a position to take action, such as state epidemiologists and local cancer control specialists. This audience often needs to obtain statistical data quickly for rapid response to health problems and cannot be expected to have the technical expertise to understand the statistical detail underlying the methods. Statisticians must consider this audience and design maps and reports in such a way as to be easily accessible by them. A forthcoming article in the International Journal of Health Geographics by Bell, Hoskins, and Wartenberg (Table 2) addresses these issues in further detail

## Conclusion

In closing, participants in the NCI workshop addressed a wide range of topics summarized in Tables 1 and 2. Initial reports address data issues, communication of results, and current limitations and areas for further development. Further discussions examined and reviewed several technical aspects of analysis relating to public health surveillance, cluster detection and spatial models, and additional reports are in preparation. We hope that public health professionals, geographers, epidemiologists, environmental scientists, and statisticians faced with the analysis of georeferenced health data find these articles to be useful as an introduction to current methods and concerns in the area.
